# Long-term health outcomes and quality of life in women with untreated pelvic floor dysfunction: a single-center cohort study

**DOI:** 10.3389/fpubh.2024.1495679

**Published:** 2025-01-07

**Authors:** Wenchao Chen, Jiang Gong, Mingsheng Liu, Ying Chang Cai

**Affiliations:** Department of Colorectal Surgery, The Quzhou Affiliated Hospital of Wenzhou Medical University, Quzhou People's Hospital, Quzhou, Zhejiang, China

**Keywords:** pelvic floor dysfunction, quality of life, sleep apnea, menopause, public health

## Abstract

**Objective:**

This study aimed to evaluate the long-term health outcomes and quality of life (HRQoL) associated with untreated pelvic floor dysfunction (PFD) in women, and to identify key factors contributing to symptom severity.

**Methods:**

A cohort of 1,651 women aged 18 years and older with untreated PFD were recruited between June 2018 and August 2023. Data on sociodemographic, lifestyle factors, and clinical history were collected via questionnaires and clinical assessments. The Pelvic Floor Distress Inventory (PFDI-20) and Short Form-12 (SF-12) were used to assess symptom severity and HRQoL.

**Results:**

The study found that 56% of participants experienced urinary incontinence (UI), 52% had pelvic organ prolapse (POP), and 47% reported gynecological disorders. Sleep apnea (OR: 18.3, *p* < 0.001) and menopause (OR: 2.48, *p* < 0.001) were significantly associated with higher symptom severity, while postpartum complications had a protective effect (OR: 0.01, *p* < 0.001). Obesity was inversely associated with symptom severity (OR: 0.3, *p* < 0.001). HRQoL scores showed that 97.6% of participants had high physical functioning (mean PF: 67.36 ± 11.62), but vitality (VT) was notably lower, with 27.8% scoring below 50 (mean VT: 54.84 ± 6.60). FI (*p* = 0.006) and pelvic pain (*p* = 0.001) were linked to significantly poorer physical functioning and higher bodily pain.

**Conclusion:**

Untreated PFD has a profound impact on HRQoL, especially in women with sleep apnea, menopause, and pelvic pain. Early diagnosis and intervention are critical to mitigating these effects and improving long-term outcomes. These findings highlight the urgent need for targeted public health strategies to address untreated PFD.

## 1 Introduction

Pelvic floor dysfunction (PFD) encompasses a range of conditions affecting the pelvic floor muscles and connective tissues, leading to compromised support of pelvic organs such as the bladder, uterus, and rectum (1, 2). Among the primary clinical manifestations are pelvic organ prolapse (POP), urinary incontinence (UI), fecal incontinence (FI), and chronic pelvic pain (CPP) (2, 3). Despite its high prevalence, especially among aging and postpartum women, PFD often remains undiagnosed and untreated. The long-term public health outcomes of untreated PFD have serious implications, particularly when considering its significant impact on the quality of life, psychological wellbeing, and healthcare burden (3, 4). Moreover, untreated PFD can lead to severe complications, including irreversible damage to pelvic organs, recurring infections, and chronic disability (4, 5).

In China, PFD is emerging as a critical public health issue due to an aging population and increasing life expectancy (6). China's rapid economic growth, combined with a declining birth rate and the aftereffects of the one-child policy, has created a demographic shift that emphasizes the healthcare needs of older women (7, 8). Studies estimate that by 2050, over 30% of China's population will be aged 60 or above, with a significant proportion of these individuals at risk for pelvic floor disorders (8, 9). Despite the growing incidence, awareness and treatment options for PFD remain limited, particularly in rural and underserved regions (10, 11). The social and cultural barriers to seeking treatment, coupled with a lack of trained healthcare professionals, further exacerbate this issue (11, 12).

The epidemiological burden and clinical sequelae of untreated PFD constitute a significant global health concern with substantial implications for female morbidity and HRQoL metrics (13). PFD manifests as a heterogeneous spectrum of clinical entities, encompassing UI, POP, FI, and pelvic-perineal pain syndromes, with varying degrees of symptom overlap and severity (13, 14). Contemporary epidemiological data demonstrate high prevalence rates across international populations, with marked diagnostic and therapeutic disparities observed in resource-limited settings, particularly throughout the Asia-Pacific region (14, 15).

Regional epidemiological analyses from Asia indicate a substantial disease burden, with cross-sectional studies documenting that ~35–45% of women experience at least one form of PFD (15). Specifically within Chinese populations, systematic reviews have reported prevalence rates of 25–40% for POP and 20–35% for UI, with associated significant decrements in validated quality of life measures (16, 17). The socioeconomic impact extends beyond individual health outcomes, manifesting in increased healthcare utilization patterns, elevated direct medical costs, and documented reductions in workforce productivity and labor force participation (17, 18).

Epidemiological data reveal distinct prevalence patterns across PFD subtypes, with UI affecting 55.8% of study participants, while FI, symptomatic uterine prolapse, and pelvic pain demonstrate rates of 10.4%, 14.0%, and 18.7%, respectively (19). These stratified prevalence data, coupled with projected demographic shifts and increasing life expectancy, underscore the imperative for evidence-based preventive interventions and standardized treatment algorithms to address the escalating burden of PFD on healthcare systems (20, 21).

In China, a study examining the knowledge, attitudes, and practices of women of childbearing age regarding PFD and pelvic floor ultrasound found that there was a significant gap in knowledge and awareness about these conditions (22). These identified knowledge gaps correlate with delayed healthcare-seeking behaviors and subsequent diagnostic delays, potentially exacerbating clinical outcomes in untreated PFD cohorts (23, 24). Despite documented high prevalence rates, longitudinal public health implications of PFD remain insufficiently characterized, particularly within rapidly aging demographic contexts such as China. Current epidemiological data demonstrate notable limitations regarding the temporal relationship between untreated PFD and its impact on HRQoL metrics and healthcare resource utilization patterns. Despite documented high prevalence rates, longitudinal implications of PFD remain incompletely characterized, particularly in rapidly aging populations. Through rigorous examination of these multifactorial relationships, this study aims to generate evidence-based insights to inform clinical intervention strategies, enhance early detection protocols, and guide public health policy development for at-risk populations. Our methodological approach emphasizes identification of modifiable risk factors and optimization of resource allocation for PFD management within contemporary healthcare systems.

## 2 Methodology

### 2.1 Study design and setting

This single-center, observational cohort study was conducted between June 2018 and August 2023 in China. The primary objective was to investigate the long-term health outcomes associated with untreated PFD in women. Eligible participants were women aged 18 years and older who presented with symptoms indicative of PFD, including UI, FI, POP, and pelvic pain. Exclusion criteria encompassed women who had received prior treatment for PFD, were pregnant, or had given birth within the previous 12 months. Additionally, women with cognitive impairments that could potentially interfere with accurate data collection were excluded.

To ensure sufficient statistical power for detecting significant associations between PFD and the public health outcomes of interest, a sample size of 1,651 women was recruited. This calculation was based on a 95% confidence level, a 3% margin of error, and a projected 25% prevalence of PFD in the population (19, 25). Anticipating a 10% dropout rate, recruitment efforts were conducted through healthcare centers, women's organizations, and online platforms. All participants provided informed consent prior to study enrollment.

### 2.2 Study population and sample size

Data collection occurred at baseline and during follow-up assessments at up to 12 months. Participants' sociodemographic characteristics, clinical history, and lifestyle factors were recorded using validated, pre-tested questionnaires administered by trained healthcare personnel. The primary variables of interest included:

- **Sociodemographic data**: age, body mass index (BMI), marital status (married, single, divorced, or widowed), employment sector (administration, industry, public service), and income level (categorized as higher, higher middle, lower middle, or middle income).
- **Lifestyle factors**: smoking habits (yes or no), frequency of alcohol consumption (daily, frequent, occasional, or never), physical activity levels (assessed through the International Physical Activity Questionnaire, IPAQ), and sleep quality (evaluated using the Pittsburgh Sleep Quality Index, PSQI).
- **Obstetric and gynecological history**: number of pregnancies, type of delivery (vaginal or instrumental), menopausal status, postpartum complications (perineal tears, pelvic floor trauma, uterine prolapse, and postpartum hemorrhage), and history of gynecological disorders (endometriosis, uterine fibroids, and ovarian cysts).
- **PFD**: the Pelvic Floor Distress Inventory (PFDI-20) was employed to evaluate the severity of PFD symptoms across three domains: POP (POPDI-6), colorectal-anal symptoms (CRADI-8), and urinary symptoms (UDI-6).

### 2.3 Clinical assessments

Physical examination employed standardized protocols encompassing POP-Q staging, Q-tip testing for urethral mobility, cough stress testing, and dynamic Valsalva maneuvers. All assessments adhered to validated clinical guidelines ensuring diagnostic consistency and reproducibility through examiner standardization protocols.

### 2.4 Quality of life measurement

HRQoL was assessed using the Short Form-12 (SF-12) questionnaire (26), measuring both physical health (physical functioning, role physical, bodily pain, general health) and mental health (vitality, social functioning, role emotional, mental health) on a 0-100 scale, with higher scores indicating better health status. Study instruments also included the Pelvic Floor Distress Inventory (PFDI-20) (27) and Pittsburgh Sleep Quality Index (PSQI) (28). In addition, CPP was assessed using the Visual Analog Scale (VAS), a validated instrument widely utilized in clinical research for quantifying pain intensity. Scores ranged from 0 (no pain) to 10 (worst possible pain), with participants marking their perceived pain severity along a 10 cm line. The use of VAS in this study ensured standardization and reproducibility in assessing pain intensity, which is crucial for comparing outcomes across similar clinical studies.

### 2.5 Outcome measures

The primary measures were:

- **Prevalence of PFD**: defined based on self-reported symptoms corroborated by clinical assessments, including UI, FI, POP, and pelvic pain.
- **Impact on quality of life**: assessed using SF-12 scores, measuring both physical and mental health dimensions.
- **Long-term complications**: included the recurrence of PFD symptoms, development of new symptoms, and other associated morbidities over time.
- **Healthcare utilization**: monitored through the frequency of medical consultations, hospitalizations, and any surgical interventions related to PFD throughout the study period.

### 2.6 Statistical analysis

The data for this study were analyzed using a combination of descriptive and inferential statistical methods. Continuous variables, such as age and body mass index (BMI), were summarized as means with standard deviations, while categorical variables, including civil status and employment sector, were presented as frequencies and percentages. For the univariate analysis, independent *t*-tests were employed to assess associations between continuous variables and PFD, while chi-square tests were applied to categorical variables. Key outcomes, such as the severity of PFD symptoms measured by the Pelvic Floor Distress Inventory (PFDI-20) subscales—POPDI-6, CRADI-8, and UDI-6—and quality of life scores from the SF-12 questionnaire, were evaluated by calculating mean differences (MD) with 95% confidence intervals (CIs).

Multivariate binary regression models controlled confounding factors in examining relationships between sociodemographic, lifestyle, and clinical variables with PFD outcomes, providing adjusted mean differences (MDs) and 95% confidence intervals (CIs). Models incorporated interaction terms for age, BMI, and menopause status. Longitudinal changes were assessed using repeated measures ANOVA, with Friedman tests employed for non-normally distributed data. Model interaction effects were tested through regression analyses to examine how factors like age and menopause modify PFD-outcome relationships (*p* < 0.05). Sensitivity analyses assessed result robustness, examining missing data patterns and parametric test assumptions. When distributional assumptions were violated, bootstrapping generated reliable standard error and confidence interval estimates, enabling precise characterization of PFD progression determinants.

## 3 Results

The study included 1,651 women with a mean age of 45 years (±15) and an average BMI of 25.0 (±4.7). Most participants were married (68%), while 23% were single. In terms of employment, self-employed women made up the largest group (16%), followed by those in administration (15%) and public service (15%). Higher middle-income (36%) was the most common income category. Non-smokers accounted for 87%, and 54% reported occasional alcohol consumption. Regarding health, sexual dysfunction affected 47% of women, sleep apnea was prevalent in 54%, and 65% reported postpartum complications. Menopause occurred in 29%, and 80% had two or more vaginal births. Obesity was present in 49%, while 47% had gynecological disorders and 37% had experienced urinary tract infections. FI was observed in 11% of participants, while 36% reported CPP, highlighting the significant burden of these conditions. Cardiovascular illnesses affected 42%, and 36% reported obstetric complications. UI was noted in 56%, and POP in 52%. HRQoL scores showed mean values of 67 (±12) for physical functioning, 60 (±10) for role physical, and 63 (±10) for bodily pain. Mental health had a mean score of 66 (±13). The physical component summary had a mean of 68.8 (±4.1), and the mental component summary was 65.17 (±2.74), reflecting overall good quality of life despite prevalent health issues, as shown in Table 1.

**Table 1:** Sociodemographic, lifestyle, and clinical characteristics of the study population (*N* = 1,651), with mean values and percentages presented for key variables.

**Variable**	***N* = 1,651^a^**
Age	45 ± 15
BMI	25.0 ± 4.7
**Civil status**	
Divorced	84 (5.1%)
Married	1,116 (68%)
Single	375 (23%)
Widowed	76 (4.6%)
**Employment sector**	
Administration	240 (15%)
Commerce	231 (14%)
Industry	223 (14%)
Public servant	242 (15%)
Retired	233 (14%)
Self-employed	257 (16%)
Student	225 (14%)
**Income level**	
Higher income	335 (20%)
Higher middle income	596 (36%)
Lower middle income	489 (30%)
Middle income	231 (14%)
**Smoking habit**	
No	1,433 (87%)
Yes	218 (13%)
**Alcohol consumption**	
Daily	25 (1.5%)
Frequently	175 (11%)
Never	377 (23%)
Occasionally	897 (54%)
Only weekends	177 (11%)
**Sexual dysfunction**	
No	879 (53%)
Yes	772 (47%)
**Sleep apnea**	
No	760 (46%)
Yes	891 (54%)
**Postpartum complications**	
No	574 (35%)
Yes	1,077 (65%)
**Menopause**	
No	1,175 (71%)
Yes	476 (29%)
**Vaginal birth**	
One	333 (20%)
Two or more	1,318 (80%)
**Instrumental birth**	
No	1,195 (72%)
Yes	456 (28%)
**Obesity**	
No	849 (51%)
Yes	802 (49%)
**Gynecological disorders**	
No	872 (53%)
Yes	779 (47%)
**Urinary tract infection**	
No	1,035 (63%)
Yes	616 (37%)
**Obstetric history**	
No	1,063 (64%)
Yes	588 (36%)
**Cardiovascular**	
No	953 (58%)
Yes	698 (42%)
**Respiratory**	
No	1,353 (82%)
Yes	298 (18%)
**Endocrine**	
No	1,364 (83%)
Yes	287 (17%)
**Urinary incontinence**	
No	722 (44%)
Yes	929 (56%)
**Fecal incontinence**	
No	1,476 (89%)
Yes	175 (11%)
**Prolapse**	
No	785 (48%)
Yes	866 (52%)
**Chronic pelvic pain**	
No	1,056 (64%)
Yes	595 (36%)
SF-12–Physical Functioning (PF)	67 ± 12
SF-12–Role Physical (RP)	60 ± 10
SF-12–Bodily Pain (BP)	63 ± 10
SF-12–General Health (GH)	65 ± 7
SF-12–Vitality (VT)	54.8 ± 6.6
SF-12–Social Functioning (SF)	68 ± 10
SF-12–Role Emotional (RE)	60 ± 7
SF-12–Mental Health (MH)	66 ± 13
PCS Physical Component Summary (PCS)	68.8 ± 4.1
Mental Component Summary (MCS)	65.17 ± 2.74
SPF-12 total	64.2 ± 3.8

^a^Mean ± SD; n (%).

The binary logistic regression analysis identified several key health conditions associated with the prolapse. Sleep apnea had a strong positive association with prolapse (OR: 18.3, 95% CI: 5.29–87.0, *p* < 0.001), indicating a much higher likelihood of the outcome. Conversely, postpartum complications showed a significant protective effect (OR: 0.01, 95% CI: 0.00–0.02, *p* < 0.001). Menopausal status demonstrated significant association with PFD, as menopausal women exhibited increased odds of developing prolapse (OR: 2.48; 95% CI: 1.58-3.93; *p* < 0.001). Obesity was inversely associated with the prolapse, suggesting a protective effect (OR: 0.3, 95% CI: 0.17–0.50, *p* < 0.001). Gynecological disorders increased the odds of prolapse (OR: 1.74, 95% CI: 1.02–2.97, *p* = 0.042). Additionally, having two or more vaginal births raised the risk (OR: 1.49, 95% CI: 1.04–2.13, *p* = 0.028), as shown in Table 2.

**Table 2:** Comparison of sociodemographic, clinical characteristics, and quality of life outcomes between women with and without POP, along with binary logistic regression results, odds ratios (OR), and 95% confidence intervals (95% CI).

**Characteristics**	***N***	**No, *N* = 785^a^**	**Yes, *N* = 8,661**	**Binary logistic regression**			
				**Coefficient**	**OR** ^b^	**95% CI** ^b^	***p*** **-value**
Age	1,651	45 ± 14	44 ± 15	0	1	0.99, 1.01	0.9
BMI	1,651	25.1 ± 4.7	24.9 ± 4.7	0.01	1.01	0.98, 1.04	0.4
Civil status	1,651						
Divorced		28 (3.6%)	56 (6.5%)	—	—	—	
Married		534 (68%)	582 (67%)	0.28	1.32	0.68, 2.60	0.4
Single		183 (23%)	192 (22%)	0.3	1.36	0.66, 2.79	0.4
Widowed		40 (5.1%)	36 (4.2%)	0.83	2.29	0.90, 5.92	0.084
Employment sector	1,651						
Administration		112 (14%)	128 (15%)	—	—	—	
Commerce		118 (15%)	113 (13%)	0.79	2.19	1.29, 3.75	0.004
Industry		110 (14%)	113 (13%)	0.42	1.53	0.89, 2.61	0.12
Public servant		129 (16%)	113 (13%)	0.45	1.57	0.93, 2.66	0.093
Retired		96 (12%)	137 (16%)	0.15	1.16	0.69, 1.98	0.6
Self-employed		115 (15%)	142 (16%)	0.29	1.34	0.80, 2.25	0.3
Student		105 (13%)	120 (14%)	0.24	1.27	0.74, 2.17	0.4
Income level	1,651						
Higher income		160 (20%)	175 (20%)	—	—	—	
Higher middle income		281 (36%)	315 (36%)	−0.11	0.89	0.60, 1.33	0.6
Lower middle income		240 (31%)	249 (29%)	0.05	1.05	0.70, 1.58	0.8
Middle income		104 (13%)	127 (15%)	−0.28	0.75	0.46, 1.24	0.3
Smoking habit	1,651						
No		677 (86%)	756 (87%)	—	—	—	
Yes		108 (14%)	110 (13%)	0.34	1.4	0.93, 2.14	0.11
Alcohol consumption	1,651						
Daily		10 (1.3%)	15 (1.7%)	—	—	—	
Frequently		81 (10%)	94 (11%)	0.15	1.16	0.36, 3.89	0.8
Never		175 (22%)	202 (23%)	−0.03	0.98	0.31, 3.11	>0.9
Occasionally		435 (55%)	462 (53%)	0.1	1.1	0.36, 3.43	0.9
Only weekends		84 (11%)	93 (11%)	0.35	1.42	0.43, 4.76	0.6
Sexual dysfunction	1,651						
No		436 (56%)	443 (51%)	—	—	—	
Yes		349 (44%)	423 (49%)	0.03	1.03	0.51, 2.06	>0.9
Sleep apnea	1,651						
No		406 (52%)	354 (41%)	—	—	—	
Yes		379 (48%)	512 (59%)	2.9	18.3	5.29, 87.0	< 0.001
Postpartum complications	1,651						
No		406 (52%)	168 (19%)	—	—	—	
Yes		379 (48%)	698 (81%)	−5.2	0.01	0.00, 0.02	< 0.001
Menopause	1,651						
No		554 (71%)	621 (72%)	—	—	—	
Yes		231 (29%)	245 (28%)	0.91	2.48	1.58, 3.93	< 0.001
Vaginal birth	1,651						
One		141 (18%)	192 (22%)	—	—	—	
Two or more		644 (82%)	674 (78%)	0.4	1.49	1.04, 2.13	0.028
Instrumental birth	1,651						
No		582 (74%)	613 (71%)	—	—	—	
Yes		203 (26%)	253 (29%)	0.04	1.05	0.76, 1.44	0.8
Obesity	1,651						
No		442 (56%)	407 (47%)	—	—	—	
Yes		343 (44%)	459 (53%)	−1.2	0.3	0.17, 0.50	< 0.001
Gynecological disorders	1,651						
No		442 (56%)	430 (50%)	—	—	—	
Yes		343 (44%)	436 (50%)	0.55	1.74	1.02, 2.97	0.042
Urinary tract infection	1,651						
No		541 (69%)	494 (57%)	—	—	—	
Yes		244 (31%)	372 (43%)	−2.4	0.09	0.06, 0.15	< 0.001
Obstetric history	1,651						
No		491 (63%)	572 (66%)	—	—	—	
Yes		294 (37%)	294 (34%)	2.1	8.45	5.16, 14.1	< 0.001
Cardiovascular	1,651						
No		462 (59%)	491 (57%)	—	—	—	
Yes		323 (41%)	375 (43%)	−0.13	0.88	0.63, 1.23	0.4
Respiratory	1,651						
No		710 (90%)	643 (74%)	—	—	—	
Yes		75 (9.6%)	223 (26%)	−1.1	0.34	0.22, 0.52	< 0.001
Endocrine	1,651						
No		651 (83%)	713 (82%)	—	—	—	
Yes		134 (17%)	153 (18%)	−0.13	0.88	0.60, 1.29	0.5
Urinary incontinence	1,651						
No		337 (43%)	385 (44%)	—	—	—	
Yes		448 (57%)	481 (56%)	0.03	1.03	0.78, 1.38	0.8
Fecal incontinence	1,651						
No		714 (91%)	762 (88%)	—	—	—	
Yes		71 (9.0%)	104 (12%)	−0.25	0.78	0.49, 1.23	0.3
Chronic pelvic pain	1,651						
No		480 (61%)	576 (67%)	—	—	—	
Yes		305 (39%)	290 (33%)	0.27	1.31	0.93, 1.84	0.12
PFDI-20–POPDI-6	1,651			0.05	1.05	0.05, 22.2	>0.9
PFDI-20–CRADI-8		111 (14%)	148 (17%)	−1.3	0.29	0.03, 2.92	0.3
PFDI-20–UDI-6		136 (17%)	142 (16%)	−0.28	0.75	0.04, 14.7	0.9
IPAQ–Physical Activity		127 (16%)	168 (19%)	0	1	1.00, 1.00	0.8
PSQI–Sleep Quality		155 (20%)	139 (16%)	0.07	1.08	0.99, 1.17	0.085
SF-12–Physical Functioning (PF)	1,651	67 ± 12	68 ± 11	−0.02	0.98	0.97, 1.00	0.038
SF-12–Role Physical (RP)	1,651	62 ± 9	58 ± 10	0.02	1.02	1.00, 1.04	0.024
SF-12–Bodily Pain (BP)	1,651	65 ± 10	62 ± 11	−0.01	1	0.98, 1.01	0.6
SF-12–General Health (GH)	1,651	65 ± 7	66 ± 7	−0.01	0.99	0.97, 1.01	0.3
SF-12–Vitality (VT)	1,651	54.9 ± 6.6	54.7 ± 6.6	−0.03	0.97	0.94, 0.99	0.01
SF-12–Social Functioning (SF)	1,651	70 ± 7	66 ± 12	0.02	1.02	0.99, 1.04	0.2
SF-12–Role Emotional (RE)	1,651	61 ± 5	58 ± 8	0	1	0.97, 1.03	0.9
SF-12–Mental Health (MH)	1,651	63 ± 15	69 ± 12	−0.15	0.86	0.84, 0.88	< 0.001
PCS Physical Component Summary (PCS)	1,651	68.9 ± 4.0	68.7 ± 4.1	−0.24	0.79	0.74, 0.84	< 0.001
Mental Component Summary (MCS)	1,651	65.17 ± 2.76	65.17 ± 2.73	−0.09	0.92	0.86, 0.98	0.01
SPF-12 total	1,651	64.9 ± 3.6	63.5 ± 3.8	0.61	1.84	1.65, 2.05	< 0.001

^a^Mean ± SD; n (%).

^b^OR, Odds Ratio; CI, Confidence Interval.

Physical Functioning had a high mean score of 67.36 (SD = 11.62), with 97.6% of participants scoring ≥50, indicating strong physical capability. Role Physical and Bodily Pain had similarly high percentages of participants scoring ≥50 (90.4% and 95.3%, respectively), reflecting minimal physical limitations or pain. Vitality, however, showed lower scores, with 27.8% scoring < 50, suggesting that a significant portion of participants experienced lower energy levels. Mental health challenges were also notable, as 15.7% scored < 50 in the Mental Health domain. The SPF-12 Total score reflected a generally high overall quality of life, with 100% of participants scoring ≥50, as shown in Table 3.

**Table 3:** Distribution of SF-12 health-related quality of life domain scores, showing mean values with standard deviations, and the percentage of participants scoring above or below 50 for each domain.

	**Mean (SD)**	**≥50 count (%)**	** < 50 count (%)**
SF-12–Physical Functioning (PF)	67.36 (11.62)	1,611 (97.6%)	40 (2.4%)
SF-12–Role Physical (RP)	59.57 (9.86)	1,492 (90.4%)	159 (9.6%)
SF-12–Bodily Pain (BP)	63.47 (10.46)	1,574 (95.3%)	77 (4.7%)
SF-12–General Health (GH)	65.49 (7.09)	1,611 (97.6%)	40 (2.4%)
SF-12–Vitality (VT)	54.84 (6.60)	1,192 (72.2%)	459 (27.8%)
SF-12–Social Functioning (SF)	67.88 (10.04)	1,527 (92.5%)	124 (7.5%)
SF-12–Role Emotional (RE)	59.51 (7.15)	1,527 (92.5%)	124 (7.5%)
SF-12–Mental Health (MH)	65.79 (13.41)	1,391 (84.3%)	260 (15.7%)
SPF-12 total	62.99 (3.83)	1,651 (100.0%)	0 (0.0%)

The comparison of various HRQoL domains across six health conditions (UI, FI, prolapse, pelvic pain, urinary tract infection, and obstetric history) revealed significant findings. For Physical Functioning, notable differences were observed for FI (*p* = 0.006), pelvic pain (*p* = 0.001), and obstetric history (*p* < 0.001). Similarly, Role Physical (RP) was significantly impacted by prolapse (*p* < 0.001), urinary tract infection (*p* < 0.001), and obstetric history (*p* < 0.001). Additionally, Bodily Pain showed significant variations for prolapse (*p* < 0.001) and urinary tract infection (*p* < 0.001), while General Health (GH) was significantly different for FI (*p* = 0.011), prolapse (*p* = 0.032), pelvic pain (*p* < 0.001), and urinary tract infections (*p* = 0.006), as shown in Table 4.

**Table 4:** Comparison of SF-12 health-related quality of life domains across various health conditions, including urinary and FI, prolapse, pelvic pain, urinary tract infection, and obstetric history, with mean scores, standard deviations, and *p*-values for each condition.

	**Urinary incontinence**			**Fecal incontinence**			**Prolapse**			**Chronic pelvic pain**			**Urinary tract infection**			**Obstetric history**		
	**No (mean** ±**SD)**	**Yes (mean** ±**SD)**	***p*** **-value**	**No (mean** ±**SD)**	**Yes (mean** ±**SD)**	***p*** **-value**	**No (mean** ±**SD)**	**Yes (mean** ±**SD)**	***p*** **-value**	**No (mean** ±**SD)**	**Yes (mean** ±**SD)**	***p*** **-value**	**No (mean** ±**SD)**	**Yes (mean** ±**SD)**	***p*** **-value**	**No (mean** ±**SD)**	**Yes (mean** ±**SD)**	***p*** **-value**
SF-12–Physical Functioning (PF)	67.16 ± 11.74	67.52 ± 11.54	0.530	67.09 ± 11.62	69.63 ± 11.39	0.006	66.81 ± 12.17	67.86 ± 11.09	0.067	68.06 ± 12.14	66.11 ± 10.54	0.001	67.73 ± 11.97	66.73 ± 11.00	0.090	65.29 ± 10.89	71.11 ± 11.97	0.000
SF-12–Role Physical (RP)	59.74 ± 9.95	59.44 ± 9.79	0.531	59.69 ± 9.82	58.59 ± 10.18	0.166	61.78 ± 8.67	57.57 ± 10.44	0.000	59.54 ± 9.72	59.62 ± 10.11	0.875	58.77 ± 10.36	60.91 ± 8.81	0.000	60.46 ± 8.95	57.97 ± 11.16	0.000
SF-12–Bodily Pain (BP)	63.24 ± 10.33	63.66 ± 10.57	0.421	63.51 ± 10.41	63.20 ± 10.91	0.713	64.88 ± 9.89	62.21 ± 10.81	0.000	63.10 ± 9.82	64.14 ± 11.49	0.053	64.42 ± 10.28	61.89 ± 10.58	0.000	63.57 ± 10.73	63.30 ± 9.96	0.612
SF-12–General Health (GH)	65.49 ± 6.96	65.48 ± 7.19	0.978	65.64 ± 7.00	64.21 ± 7.65	0.011	65.10 ± 7.38	65.84 ± 6.79	0.032	64.55 ± 7.39	67.15 ± 6.17	0.000	65.85 ± 7.19	64.87 ± 6.87	0.006	65.28 ± 7.25	65.86 ± 6.77	0.113
SF-12–Vitality (VT)	54.90 ± 6.63	54.78 ± 6.57	0.724	54.82 ± 6.59	54.94 ± 6.65	0.820	54.94 ± 6.58	54.74 ± 6.61	0.559	55.00 ± 6.65	54.54 ± 6.50	0.174	54.38 ± 6.52	55.60 ± 6.66	0.000	54.99 ± 6.67	54.55 ± 6.46	0.188
SF-12–Social Functioning (SF)	67.95 ± 10.22	67.84 ± 9.90	0.820	68.02 ± 9.96	66.73 ± 10.65	0.106	70.09 ± 6.80	65.89 ± 11.91	0.000	67.01 ± 10.57	69.44 ± 8.81	0.000	67.97 ± 10.40	67.73 ± 9.39	0.637	70.95 ± 6.35	62.34 ± 12.74	0.000
SF-12–Role Emotional (RE)	59.40 ± 7.13	59.59 ± 7.17	0.596	59.45 ± 7.24	59.99 ± 6.36	0.341	61.31 ± 4.86	57.87 ± 8.39	0.000	59.69 ± 7.30	59.19 ± 6.88	0.176	60.38 ± 5.77	58.05 ± 8.82	0.000	58.45 ± 7.87	61.42 ± 5.11	0.000
SF-12–Mental Health (MH)	65.72 ± 13.48	65.84 ± 13.36	0.853	65.62 ± 13.43	67.19 ± 13.14	0.144	62.78 ± 14.67	68.51 ± 11.51	0.000	66.63 ± 13.52	64.29 ± 13.08	0.001	64.89 ± 14.02	67.29 ± 12.17	0.000	64.44 ± 14.09	68.23 ± 11.70	0.000
SPF-12 total	64.11 ± 3.76	64.18 ± 3.76	0.685	64.09 ± 3.70	64.65 ± 4.18	0.061	64.88 ± 3.59	63.49 ± 3.79	0.000	64.21 ± 3.56	64.04 ± 4.10	0.387	64.04 ± 3.89	64.33 ± 3.53	0.137	64.04 ± 3.82	64.35 ± 3.64	0.112

The PFDI−20 scores demonstrated significant symptom improvements between baseline and 12–month follow–up assessments. The POPDI−6 measure decreased from baseline (mean: 45.3 ± 22.1) to 12 months (mean: 43.2 ± 21.8), with a mean difference (MD) of −2.10 (95% CI: −5.93 to 1.72, *p* = 0.01), indicating a reduction in symptoms. Similarly, the CRADI−8 scores improved from baseline (mean: 34.6 ± 19.2) to 12 months (mean: 32.4 ± 18.6), with an MD of −2.19 (95% CI: −5.15 to 0.78, *p* = 0.02). The total PFDI−20 score also showed significant improvement, decreasing from baseline (mean: 89.4 ± 35.6) to 12 months (mean: 85.5 ± 34.8), with an MD of −3.89 (95% CI: −14.43 to 6.65, *p* = 0.03). However, no significant changes were observed in Physical Activity scores (baseline: 7.2 ± 2.8; 12 months: 7.4 ± 2.9; *p* = 0.116) or Sleep Quality measures (baseline: 6.8 ± 3.1; 12 months: 6.9 ± 3.0; *p* = 0.866) over the follow–up period. These findings are summarized in Table 5.

**Table 5:** Mean differences (MD), 95% confidence intervals (CI), and *p*-values for PFDI-20 subscales, total PFDI-20 score, IPAQ, and PSQI, highlighting significant improvements in PFDI-20 scores.

	**MD (95% CI)**	***p*-value**
PFDI-20–POPDI-6	−2.10 (−5.93 to 1.72)	0.01
PFDI-20–CRADI-8	−2.19 (−5.15 to 0.78)	0.02
PFDI-20–UDI-6	0.40 (−3.35 to 4.15)	0.02
Total PFDI-20	−3.89 (−14.43 to 6.65)	0.03
IPAQ–Physical Activity	0.00 (−0.00 to 0.00)	0.116
PSQI–Sleep Quality	0.01 (−0.10 to 0.12)	0.866

In the bivariable analysis, SF−12 Physical Functioning remained significant (MD: 0.15, 95% CI: 0.037 to 0.267, *p* = 0.009), and being in the industry sector had a negative association (MD: −4.01, 95% CI: −7.91 to −0.11, *p* = 0.043). Sleep Apnea (Yes) (MD: −7.45, 95% CI: −10.11 to −4.80, *p* < 0.001) and Postpartum complications (Yes) (MD: −11.58, 95% CI: −14.33 to −8.83, *p* < 0.001) were strongly associated with the outcome, and Pelvic pain (Yes) was positively associated (MD: 2.85, 95% CI: 0.08 to 5.63, *p* = 0.044). However, In the multivariable analysis, SF−12 Physical Functioning remained significant (MD: 0.16, 95% CI: 0.031 to 0.281, *p* = 0.015), and the negative association with Industry employment persisted (MD: −5.58, 95% CI: −10.32 to −0.85, *p* = 0.021). Strong positive associations were observed for Sexual Dysfunction (Yes) (MD: 15.74, 95% CI: 9.45 to 22.03, *p* < 0.001), Sleep Apnea (Yes) (MD: −14.80, 95% CI: −21.50 to −8.11, *p* < 0.001), and Postpartum complications (Yes) (MD: −11.70, 95% CI: −16.62 to −6.77, *p* < 0.001). Menopause (Yes) was positively linked to the outcome (MD: 4.92, 95% CI: 0.90 to 8.93, *p* = 0.017), and Prolapse (Yes) (MD: −4.90, 95% CI: −7.88 to −1.93, *p* = 0.001) and Pelvic Pain (Yes) (MD: 3.93, 95% CI: 0.99 to 6.88, *p* = 0.009) remained significant, as shown in Table 6.

**Table 6:** Mean differences (MD), 95% confidence intervals (CI), and *p*-values for bivariate and multivariate analyses of key demographic and clinical variables, highlighting significant associations with health outcomes.

	**Bivariate analysis**				**Multivariate analysis**			
**Variable**	**MD**	**95% CI-Lower**	**95% CI-Upper**	***p*** **-value**	**MD**	**95% CI-Lower**	**95% CI-Upper**	***p*** **-value**
Age	0.11	0.02	0.20	0.02	0.07	−0.02	0.15	0.13
BMI	0.02	−0.26	0.31	0.87	0.12	−0.15	0.39	0.39
PFDI-20–POPDI-6	−1.55	−29.67	26.57	0.91	−2.45	−29.01	24.10	0.86
PFDI-20–CRADI-8	−19.24	−41.02	2.54	0.08	−9.88	−30.46	10.71	0.35
PFDI-20–UDI-6	11.32	−16.28	38.91	0.42	4.08	−21.88	30.04	0.76
IPAQ–Physical Activity	0.00	0.00	0.01	0.29	0.00	0.00	0.01	0.46
PSQI–Sleep Quality	0.13	−0.65	0.91	0.74	0.11	−0.63	0.84	0.78
SF-12–Physical Functioning (PF)	0.15	0.04	0.27	0.01	0.16	0.03	0.28	0.01
Civil status married	1.28	−1.57	4.13	0.38	−2.24	−8.04	3.55	0.45
Civil status single	−1.51	−4.70	1.67	0.35	−3.40	−9.59	2.80	0.28
Civil status widowed	−0.52	−6.89	5.85	0.87	−4.76	−12.89	3.36	0.25
Employment sector-commerce	0.92	−2.93	4.76	0.64	−2.39	−7.10	2.32	0.32
Employment sector-industry	−4.01	−7.91	−0.11	0.04	−5.58	−10.32	−0.85	0.02
Employment sector-public servant	−0.58	−4.35	3.20	0.76	−2.65	−7.31	2.00	0.26
Employment sector-retired	0.51	−3.32	4.34	0.79	−2.16	−6.84	2.53	0.37
Employment sector-self-employed	0.75	−2.93	4.43	0.69	−2.51	−7.07	2.06	0.28
Employment sector-student	−0.98	−4.87	2.91	0.62	−3.71	−8.43	1.01	0.12
Income level-higher middle	1.02	−1.76	3.80	0.47	3.00	−0.50	6.49	0.09
Income level-lower middle	−1.34	−4.27	1.58	0.37	1.30	−2.33	4.93	0.48
Income level-middle income	1.86	−1.98	5.71	0.34	2.04	−2.33	6.41	0.36
Smoking habit-yes	−3.32	−7.26	0.62	0.10	−3.18	−6.90	0.54	0.09
Alcohol consumption-frequently	−1.65	−5.99	2.69	0.46	2.23	−8.69	13.16	0.69
Alcohol consumption-never	0.57	−2.61	3.75	0.73	4.93	−5.59	15.46	0.36
Alcohol consumption-occasionally	−0.47	−3.15	2.21	0.73	3.78	−6.57	14.13	0.47
Alcohol consumption-only weekends	2.76	−1.56	7.07	0.21	6.99	−3.92	17.89	0.21
Sexual dysfunction-yes	−2.61	−5.28	0.07	0.06	15.74	9.45	22.03	0.00
Sleep apnea-yes	−7.45	−10.11	−4.80	0.00	−14.80	−21.50	−8.11	0.00
Postpartum complications-yes	−11.58	−14.33	−8.83	0.00	−11.70	−16.62	−6.77	0.00
Menopause-yes	0.83	−2.12	3.77	0.58	4.92	0.90	8.93	0.02
Vaginal birth-two or more	−1.55	−4.88	1.77	0.36	−1.56	−4.70	1.58	0.33
Instrumental birth-yes	−3.09	−6.08	−0.11	0.04	−2.64	−5.46	0.19	0.07
Obesity-yes	0.83	−1.85	3.50	0.54	0.86	−3.68	5.40	0.71
Gynecological disorders-yes	2.13	−0.54	4.80	0.12	4.57	−0.10	9.24	0.06
Urinary tract infection-yes	−0.33	−3.09	2.43	0.81	1.91	−1.43	5.25	0.26
Obstetric history-yes	−4.46	−7.24	−1.68	0.00	−13.25	−16.63	−9.86	0.00
Cardiovascular-yes	9.37	6.70	12.03	0.00	11.14	8.32	13.97	0.00
Respiratory-yes	0.96	−2.51	4.43	0.59	0.75	−2.97	4.46	0.69
Endocrine-yes	−0.85	−4.37	2.67	0.64	−0.70	−4.02	2.62	0.68
Urinary incontinence -yes	0.18	−2.51	2.88	0.89	0.21	−2.34	2.75	0.87
Fecal incontinence-yes	−1.90	−6.24	2.44	0.39	−0.88	−5.00	3.24	0.68
Prolapse-yes	−8.23	−10.87	−5.59	0.00	−4.90	−7.88	−1.93	0.00
Pelvic pains-yes	2.85	0.08	5.63	0.04	3.93	0.99	6.88	0.01

**Figure 1:**
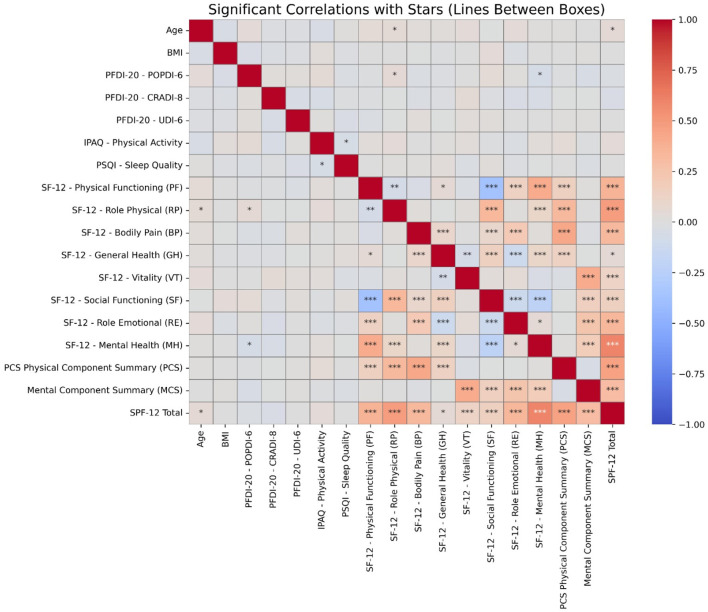
Heatmap displaying significant correlations between variables. The color intensity represents the strength and direction of the correlations. Asterisks indicate statistically significant relationships, with * denoting *p* < 0.05, ** for *p* < 0.01, and *** for *p* < 0.001. Stronger correlations are more likely to indicate clinically meaningful associations.

Correlation analysis demonstrated significant relationships between HRQoL domains and clinical variables. Age and BMI exhibited weak positive correlations with PFDI-20 subscales (POPDI-6, CRADI-8, UDI-6), indicating POP and urinary distress associations. SF-12 Physical Functioning showed strong positive correlations with Role Physical, Bodily Pain, and Mental Health components. Physical Component Summary (PCS) correlated strongly with Physical Functioning, while Mental Component Summary (MCS) demonstrated strong associations with Mental Health. PFD measures showed weaker correlations with HRQoL domains, as shown in Figure 1.

## 4 Discussion

This study provides a comprehensive analysis of the HRQoL and associated health conditions among a cohort of 1,651 women, highlighting several key findings that contribute to our understanding of PFD and its broader implications on wellbeing. The analysis of variables such as sleep apnea, postpartum complications, menopause, and obesity offers both positive and negative insights when compared to existing literature. The study reveals a high prevalence of sexual dysfunction (47%), sleep apnea (54%), and postpartum complications (65%) among the participants. These findings are consistent with previous literature, which has reported similar prevalence rates for these conditions (29, 30). For instance, a study reported that a prevalence of sexual dysfunction in 40% of women in the United States (29). Similarly, a study found that 48.3% of older adults experienced sexual dysfunction (31).

Previous research has consistently highlighted the substantial burden that pelvic floor disorders impose on women's physical and mental wellbeing. For instance studies demonstrated that POP significantly impairs physical functioning and daily activities, with a profound effect on women's self-reported quality of life (32–34). Similarly, a study reported that menopause exacerbates symptoms of PFD, leading to a decline in both physical and emotional health (35). Our findings support these conclusions by demonstrating strong associations between menopause, prolapse, and reduced HRQoL, especially in physical domains like role physical and bodily pain.

Sleep apnea and its role in PFD, while less frequently discussed, is gaining attention. Recent studies showed that women with sleep apnea tend to have worse outcomes in terms of UI and POP, which is reflected in our findings of a strong positive association between sleep apnea and lower quality of life (36, 37). The connection between sleep-disordered breathing and pelvic floor health may be mediated by several factors, including increased intra-abdominal pressure during sleep, which contributes to the worsening of pelvic floor symptoms (38–40). This is an area where further research is needed, as the interplay between sleep health and PFD remains understudied.

Interestingly, obesity has been traditionally considered a risk factor for PFD, particularly UI and POP (41). Recent studies consistently demonstrate that higher BMI is linked to greater severity of symptoms due to increased abdominal pressure and stress on pelvic tissues (42, 43). However, our study's finding of a protective effect of obesity on PFD contradicts the prevailing consensus. This discrepancy could be due to differences in population characteristics, such as access to healthcare, lifestyle interventions targeting obesity, or varying obesity phenotypes (e.g., distribution of fat mass) (44, 45). It is also possible that the protective effect observed is confounded by other variables, and further research is needed to clarify the relationship between BMI and pelvic health outcomes (46–48).

Postpartum complications are frequently cited as major contributors to long-term PFD (49). Bozkurt et al. (50) emphasizes the link between vaginal deliveries, particularly those involving instrumental assistance, and the development of conditions like UI and prolapse. In addition, further studies suggest that damage to the pelvic muscles and nerves during childbirth can have lasting effects, which contrasts with our finding that postpartum complications had a protective effect (51, 52). This could be explained by differences in the types of postpartum complications considered or by the influence of postnatal care practices, which have improved in recent years (53). However, the discrepancy highlights the need for a nuanced understanding of postpartum recovery and its long-term implications on pelvic health (54, 68).

Sexual dysfunction is a critical concern in women with pelvic floor disorders, as conditions like prolapse, incontinence, and CPP significantly impact sexual satisfaction and functioning (55, 56). Our results also suggest that sexual dysfunction is strongly associated with lower HRQoL, particularly in mental and emotional health domains. This finding reinforces the importance of addressing sexual health in the management of pelvic floor disorders, as it remains an underexplored yet significant factor affecting overall wellbeing.

The findings from your study reveal a significant prevalence of PFD symptoms among women, including UI (56%), pelvic organ prolapse (52%), and CPP (36%). These results are consistent with the literature, which indicates that PFDs are common and debilitating conditions affecting a substantial portion of the female population. For instance, a study reported that stress UI affects approximately 46% of women, while the lifetime risk of pelvic organ prolapse surgery is estimated to be between 12% and 19% (57–59). Additionally, the association of obesity with PFDs is well-documented; your study found that 49% of participants were classified as obese, aligning with findings of previous study (19), which emphasize obesity as a significant risk factor for developing UI and pelvic organ prolapse. Menopausal status also emerged as a critical factor in your results, with menopausal women exhibiting increased odds of prolapse (OR: 2.48), corroborating a study (60), who noted that hormonal changes during menopause significantly heighten the risk of female sexual dysfunction and other PFDs. Furthermore, your study's observation that 80% of participants had two or more vaginal births aligns with the literature, which highlights the increased risk of PFDs associated with multiple vaginal deliveries (59, 61–63).

PFDs represent a significant public health issue, imposing a considerable economic burden due to increased healthcare utilization, direct medical costs, and lost productivity. The prevalence of these disorders is notable, with estimates suggesting that they affect ~25% to 50% of women globally, leading to substantial healthcare demands and economic implications (25, 64, 69). Untreated PFDs often result in repeated medical consultations and a heightened risk of surgical interventions, which further exacerbates healthcare costs and resource utilization (62, 63, 70). The emotional and psychological impacts of PFDs also contribute to indirect costs, including reduced work capacity and caregiver burden, highlighting the multifaceted nature of the economic burden associated with these conditions (58).

Public health policies should prioritize subsidized screening programs and early diagnostic efforts to mitigate the long-term impacts of PFDs. Evidence suggests that timely interventions can significantly improve women's quality of life and enhance workforce participation, ultimately leading to reduced overall healthcare costs (65). Targeted measures, such as community outreach and improved access to care, are particularly critical in underserved regions where healthcare disparities may exacerbate the prevalence and consequences of untreated PFDs (66). The need for effective public health strategies is underscored by the projected increase in the prevalence of PFDs, which is expected to double from 2010 to 2050 in the United States alone, necessitating proactive measures to address this growing public health concern (67, 71).

This comprehensive cohort study elucidates the public health implications of untreated PFD through systematic evaluation of sociodemographic, lifestyle, and clinical determinants affecting symptom severity and HRQoL. Analysis revealed significant associations between PFD and comorbid conditions (sleep apnea, menopause, postpartum complications), emphasizing the necessity for early intervention strategies. These findings advance current understanding of PFD progression in an understudied population, providing evidence-based data to inform clinical protocols and public health initiatives.

Study limitations include its observational design, precluding causal relationship establishment between PFD and health outcomes, and potential reporting bias from self-reported variables (physical activity, sleep quality, PFD symptoms). Despite recruitment efforts, the cohort may not fully represent all untreated PFD populations across socioeconomic and geographic strata. Exclusion criteria (recent pregnancies, active PFD treatment) and 12-month follow-up duration limit result generalizability and long-term complication assessment. Randomized controlled trials with extended follow-up periods are needed to comprehensively evaluate PFD progression and public health impact.

## 5 Conclusion

In conclusion, this single-center cohort study demonstrates the significant impact of untreated PFD on women's health, particularly in terms of reduced HRQoL. Key factors such as sleep apnea, menopause, and postpartum complications were associated with increased symptom severity, while obesity showed a protective effect that warrants further investigation. Conditions like FI, pelvic pain, and prolapse were linked to poorer physical functioning and heightened bodily pain. Despite the prevalence of PFD, many participants maintained good overall health, indicating that early diagnosis and intervention could mitigate long-term consequences. These findings highlight the need for targeted public health strategies, improved screening, and timely treatment to reduce the burden of untreated PFD and improve women's quality of life.

## Data Availability

The original contributions presented in the study are included in the article/supplementary material, further inquiries can be directed to the corresponding author.

## References

[1] Tim S Mazur-Bialy AI. The most common functional disorders and factors affecting female pelvic floor. Life. (2021) 11:1397. 10.3390/life11121397 34947928 PMC8704638

[2] Davis K Kumar D Stanton SL. Pelvic floor dysfunction: the need for a multidisciplinary team approach. Urogynecology. (2003) 9:23–36. 10.1097/01.SPV.0000056863.70573.B0

[3] Zhoolideh P Ghaderi F Salahzadeh Z. Are there any relations between posture and pelvic floor disorders? a literature review. Cresc J Med Biol Sci. (2017) 4:4.

[4] La Rosa V Ciebiera M Lin L-T Sleiman Z Cerentini T Lordelo P. Multidisciplinary management of women with pelvic organ prolapse, urinary incontinence and lower urinary tract symptoms. A clinical and psychological overview. Menopause Review/Przeglad Menopauzalny. (2019) 18:184–90. 10.5114/pm.2019.89496 31975987 PMC6970416

[5] American College of Obstetricians and Gynecologists and the American Urogynecologic Society. Pelvic organ prolapse. Female Pelvic Med Reconstr Surg. (2019) 25(6):397-408. 10.1097/SPV.0000000000000794 31663890

[6] Ge J Yang P Zhang Y Li X Wang Q Lu Y. Prevalence and risk factors of urinary incontinence in Chinese women: a population-based study. Asia Pacific J Public Health. (2015) 27:NP1118–NP31. 10.1177/1010539511429370 22186396

[7] Alpermann B Zhan S. Population planning after the one-child policy: Shifting modes of political steering in China. J Contemp China. (2019) 28:348–66. 10.1080/10670564.2018.1542218

[8] Abrahamson P. End of an era? China's one-child policy and its unintended consequences. Asian Soc Work Policy Rev. (2016) 10:326–38. 10.1111/aswp.12101

[9] Wang B Chen Y Zhu X Wang T Li M Huang Y. Global burden and trends of pelvic organ prolapse associated with aging women: an observational trend study from 1990 to 2019. Front Public Health. (2022) 10:975829. 10.3389/fpubh.2022.975829 36187690 PMC9521163

[10] Nauheim J McKay E Laudano M Abraham N. Healthcare disparities in the treatment of pelvic floor disorders. Curr Bladder Dysfunct Rep. (2020) 15:135–41. 10.1007/s11884-020-00598-w

[11] Wynaden D Chapman R Orb A McGowan S Zeeman Z Yeak S. Factors that influence Asian communities' access to mental health care. Int J Ment Health Nurs. (2005) 14:88–95. 10.1111/j.1440-0979.2005.00364.x 15896255

[12] Lai Y-t Lin A-w Zheng Z-h Wang Y-l Yu H-h Jiang X-y. Perceptions of pelvic floor dysfunction and rehabilitation care amongst women in southeast China after radical hysterectomy: a qualitative study. BMC Women's Health. (2022) 22:108. 10.1186/s12905-022-01687-0 35397542 PMC8994321

[13] Dominiak-Felden G Cohet C Atrux-Tallau S Gilet H Tristram A Fiander A. Impact of human papillomavirus-related genital diseases on quality of life and psychosocial wellbeing: results of an observational, health-related quality of life study in the UK. BMC Public Health. (2013) 13:1–11. 10.1186/1471-2458-13-1065 24215264 PMC4225724

[14] Martínez-Galiano JM Peinado-Molina RA Martínez-Vazquez S Hita-Contreras F Delgado-Rodríguez M Hernández-Martínez A. Influence of pelvic floor disorders on sexuality in women. Int J Gyneco Obstet. (2024) 164:1141–50. 10.1002/ijgo.15189 37830235

[15] Hallock JL Handa VL. The epidemiology of pelvic floor disorders and childbirth: an update. Obstet Gynecol Clinics. (2016) 43:1–13. 10.1016/j.ogc.2015.10.008 26880504 PMC4757815

[16] Zhu Q Shu H Dai Z. Effect of pelvic floor dysfunction on sexual function and quality of life in Chinese women of different ages: an observational study. Geriatr Gerontol Int. (2019) 19:299–304. 10.1111/ggi.13618 30811813

[17] Xue K Palmer MH Zhou F. Prevalence and associated factors of urinary incontinence in women living in China: a literature review. BMC Urol. (2020) 20:1–26. 10.1186/s12894-020-00735-x 33054777 PMC7559450

[18] De Putter C Selles R Polinder S Panneman M Hovius S van Beeck EF. Economic impact of hand and wrist injuries: health-care costs and productivity costs in a population-based study. JBJS. (2012) 94:e56. 10.2106/JBJS.K.00561 22552678

[19] Peinado-Molina RA Hernández-Martínez A Martínez-Vázquez S Rodríguez-Almagro J Martínez-Galiano JM. Pelvic floor dysfunction: prevalence and associated factors. BMC Public Health. (2023) 23:2005. 10.1186/s12889-023-16901-3 37838661 PMC10576367

[20] Hutchison D Ali M Zillioux J Ortiz NM Smith R Rapp DE. Pelvic floor muscle training in the management of female pelvic floor disorders. Curr Bladder Dysfunct Rep. (2022) 17:115–24. 10.1007/s11884-022-00653-8

[21] Aschkenazi SO Goldberg RP. Female sexual function and the pelvic floor. Expert Rev Obstet Gynecol. (2009) 4:165–78. 10.1586/17474108.4.2.165

[22] Wu X Yi X Zheng X Chen Z Liu J Dai X. Knowledge, attitudes, and practice of pelvic floor dysfunction and pelvic floor ultrasound among women of childbearing age in Sichuan, China. Front Public Health. (2023) 11:1160733. 10.3389/fpubh.2023.1160733 37234767 PMC10206020

[23] Davis K Kumar D. Pelvic floor dysfunction: a conceptual framework for collaborative patient-centred care. J Adv Nurs. (2003) 43:555–68. 10.1046/j.1365-2648.2003.02754.x 12950561

[24] Elneil S Romanzi L Goh J Haylen B Grace Chen CC Ghoniem G. An International Continence Society (ICS) report on the terminology for female pelvic floor fistulas. Neurourol Urodyn. (2020) 39:2040–71. 10.1002/nau.24508 33068487

[25] Wu JM Vaughan CP Goode PS Redden DT Burgio KL Richter HE. Prevalence and trends of symptomatic pelvic floor disorders in US women. Obstet Gynecol. (2014) 123:141–8. 10.1097/AOG.0000000000000057 24463674 PMC3970401

[26] Shah CH Brown JD. Reliability and validity of the short-form 12 item version 2 (SF– 12v2) health-related quality of life survey and disutilities associated with relevant conditions in the US older adult population. J Clin Med. (2020) 9:661. 10.3390/jcm9030661 32121371 PMC7141358

[27] de Arruda GT dos Santos Henrique T Virtuoso JF. Pelvic floor distress inventory (PFDI)—systematic review of measurement properties. Int Urogynecol J. (2021) 32:2657–69. 10.1007/s00192-021-04748-4 33710430

[28] Raniti MB Waloszek JM Schwartz O Allen NB Trinder J. Factor structure and psychometric properties of the Pittsburgh Sleep Quality Index in community-based adolescents. Sleep. (2018) 41:zsy066. 10.1093/sleep/zsy066 29608755

[29] Martínez Vázquez S Hernández Martínez A Peinado Molina RA Martínez Galiano JM. Association between sexual function in women and sleep quality. Front Med. (2023) 10:1196540. 10.3389/fmed.2023.1196540 37636576 PMC10457145

[30] Shah AF Chawla I Goel K Gollen R Singh R. Impact of obesity on female sexual dysfunction: a remiss. Curr Women's Health Rev. (2021) 17:21–8. 10.2174/1573404816999200917121519

[31] Tinetti A Weir N Tangyotkajohn U Jacques A Thompson J Briffa K. Help-seeking behaviour for pelvic floor dysfunction in women over 55: drivers and barriers. Int Urogynecol J. (2018) 29:1645–53. 10.1007/s00192-018-3618-2 29552740

[32] Blomquist JL Carroll M Muñoz A Handa VL. Pelvic floor muscle strength and the incidence of pelvic floor disorders after vaginal and cesarean delivery. Am J Obstet Gynecol. (2020) 222:e1–e8. 10.1016/j.ajog.2019.08.003 31422064

[33] Ouchi M Kato K Gotoh M Suzuki S. Physical activity and pelvic floor muscle training in patients with pelvic organ prolapse: a pilot study. Int Urogynecol J. (2017) 28:1807–15. 10.1007/s00192-017-3356-x 28624919

[34] Imoto A Sarker M Akter R Matsuyama A Honda S. Health-related quality of life in parous women with pelvic organ prolapse and/or urinary incontinence in Bangladesh. Int Urogynecol J. (2021) 32:887–95. 10.1007/s00192-020-04410-5 32607714

[35] Carcelén-Fraile MdC Aibar-Almazán A Martínez-Amat A Cruz-Díaz D Díaz-Mohedo E Redecillas-Peiró MT. Effects of physical exercise on sexual function and quality of sexual life related to menopausal symptoms in peri-and postmenopausal women: A systematic review. Int J Environm Res Public Health. (2020) 17:2680. 10.3390/ijerph17082680 32295114 PMC7215442

[36] Fitzgerald MP Mulligan M Parthasarathy S. Paper 47: association of obstructive sleep apnea and nocturia, and improvement of nocturia with continuous positive airways (CPAP) treatment of OSA. Urogynecology. (2005) 11:S23. 10.1097/01.spv.0000176125.94409.8b

[37] Cameron AP Smith AR Lai HH Bradley CS Liu AB Merion RM. Bowel function, sexual function, and symptoms of pelvic organ prolapse in women with and without urinary incontinence. Neurourol Urodyn. (2018) 37:2586–96. 10.1002/nau.23587 29635702 PMC6179951

[38] Tan I-F Horne AW. Obesity and chronic pelvic pain. In: Obesity Gynecology. London: Elsevier (2020). p. 281–91.

[39] Bharucha AE. Pelvic floor: anatomy and function. Neurogastroenterol Motility. (2006) 18:507–19. 10.1111/j.1365-2982.2006.00803.x 16771766

[40] Memon HU Handa VL. Vaginal childbirth and pelvic floor disorders. Women's Health. (2013) 9:265–77. 10.2217/WHE.13.17 23638782 PMC3877300

[41] Greer WJ Richter HE Bartolucci AA Burgio KL. Obesity and pelvic floor disorders: a systematic review. Obstet Gynecol. (2008) 112:341–9. 10.1097/AOG.0b013e31817cfdde 18669733 PMC3252023

[42] Chilaka C Toozs-Hobson P Chilaka V. Pelvic floor dysfunction and obesity. Best Pract Res Clin Obstet Gynaecol. 90:102389. 10.1016/j.bpobgyn.2023.102389 37541114

[43] Laven BA Orsini N Andersson S-O Johansson J-E Gerber GS Wolk A. Birth weight, abdominal obesity and the risk of lower urinary tract symptoms in a population based study of Swedish men. J Urol. (2008) 179:1891–6. 10.1016/j.juro.2008.01.029 18353377

[44] Pratt CA Loria CM Arteaga SS Nicastro HL Lopez-Class M de Jesus JM. A systematic review of obesity disparities research. Am J Prev Med. (2017) 53:113–22. 10.1016/j.amepre.2017.01.041 28341221

[45] Bansal V Conroy C Lee J Schwartz A Tominaga G Coimbra R. Is bigger better? The effect of obesity on pelvic fractures after side impact motor vehicle crashes. J Trauma Acute Care Surg. (2009) 67:709–14. 10.1097/TA.0b013e3181af6cc1 19820575

[46] Goldberg RP Kwon C Gandhi S Atkuru LV Sorensen M Sand PK. Urinary incontinence among mothers of multiples: the protective effect of cesarean delivery. Am J Obstet Gynecol. (2003) 188:1447–53. 10.1067/mob.2003.451 12824977

[47] Schulten SF Claas-Quax MJ Weemhoff M van Eijndhoven HW van Leijsen SA Vergeldt TF. Risk factors for primary pelvic organ prolapse and prolapse recurrence: an updated systematic review and meta-analysis. Am J Obstet Gynecol. (2022) 227:192–208. 10.1016/j.ajog.2022.04.046 35500611

[48] Burns JW Quartana PJ Bruehl S Janssen I Dugan SA Appelhans B. Chronic pain, body mass index and cardiovascular disease risk factors: tests of moderation, unique and shared relationships in the Study of Women's Health Across the Nation (SWAN). J Behav Med. (2015) 38:372–83. 10.1007/s10865-014-9608-z 25427423 PMC4496954

[49] Dasikan Z Ozturk R Ozturk A. Pelvic floor dysfunction symptoms and risk factors at the first year of postpartum women: a cross-sectional study. Contemp Nurse. (2020) 56:132–45. 10.1080/10376178.2020.1749099 32216721

[50] Bozkurt M Yumru AE Sahin L. Pelvic floor dysfunction, and effects of pregnancy and mode of delivery on pelvic floor. Taiwanese J Obstet Gynecol. (2014) 53:452–8. 10.1016/j.tjog.2014.08.001 25510682

[51] Baessler K Schuessler B. Childbirth-induced trauma to the urethral continence mechanism: review and recommendations. Urology. (2003) 62:39–44. 10.1016/j.urology.2003.08.001 14550836

[52] Phillips C Monga A. Childbirth and the pelvic floor: “the gynaecological consequences”. Rev Gynaecolog Pract. (2005) 5:15–22. 10.1016/j.rigp.2004.09.002

[53] Dietz H Schierlitz L. Pelvic floor trauma in childbirth–myth or reality? Austral New Zeal J Obstet Gynaecol. (2005) 45:3–11. 10.1111/j.1479-828X.2005.00363.x 15730357

[54] O'Malley D Higgins A Smith V. Exploring the complexities of postpartum sexual health. Current Sexual Health Rep. (2021) 2021:1–8. 10.1007/s11930-021-00315-6

[55] Verbeek M Hayward L. Pelvic floor dysfunction and its effect on quality of sexual life. Sexual Med Rev. (2019) 7:559–64. 10.1016/j.sxmr.2019.05.007 31351916

[56] Achtari C Dwyer PL. Sexual function and pelvic floor disorders. Best Pract Res Clini Obstet Gynaecol. (2005) 19:993–1008. 10.1016/j.bpobgyn.2005.08.012 16185931

[57] Zhang S. The causal effect of reproductive factors on pelvic floor dysfunction: a mendelian randomization study. BMC Women S Health. (2024) 24:1. 10.1186/s12905-024-02914-6 38281950 PMC10822177

[58] Ghetti C Skoczylas LC Oliphant SS Nikolajski C Lowder JL. The emotional burden of pelvic organ prolapse in women seeking treatment. Female Pelvic Med Reconst Surg. (2015) 21:332–8. 10.1097/SPV.0000000000000190 26506161 PMC4624225

[59] Laakkonen EK Kulmala J Aukee P Hakonen H Kujala UM Lowe DA. Female reproductive factors are associated with objectively measured physical activity in middle-aged women. PLoS ONE. (2017) 12:e0172054. 10.1371/journal.pone.0172054 28225786 PMC5321412

[60] Zhuo Z Wang C Yu H Li J. The relationship between pelvic floor function and sexual function in perimenopausal women. Sexual Med. (2021) 9:100441. 10.1016/j.esxm.2021.100441 34628115 PMC8766258

[61] Hage-Fransen MAH Wiezer M Otto AD Wieffer-Platvoet M Slotman MH Maria WGN-vdS. Pregnancy- and obstetric-related risk factors for urinary incontinence, fecal incontinence, or pelvic organ prolapse later in life: a systematic review and meta-analysis. Acta Obstetricia Et Gynecologica Scandinavica. (2020) 100:373–82. 10.1111/aogs.14027 33064839

[62] Demissie BA. Prevalence and associated factors of symptomatic pelvic floor disorders among women living in Debre Tabor Town, Northwest Amhara, Ethiopia. BMC Women S Health. (2024) 24:367. 10.1186/s12905-024-03176-y 38915020 PMC11194954

[63] Molina RAP. Influence of pelvic floor disorders on quality of life in women. Front Public Health. (2023) 11:1180907. 10.3389/fpubh.2023.1180907 37942254 PMC10629477

[64] Duran P Sesillo FB Cook MS Burnett LA Menefee SA Do E. Proregenerative extracellular matrix hydrogel mitigates pathological alterations of pelvic skeletal muscles after birth injury. Science Transl Med. (2023) 15:abj3138. 10.1126/scitranslmed.abj3138 37531414 PMC10460616

[65] Dheresa M Worku A Oljira L Mengiste B Assefa N Berhane Y. One in five women suffer from pelvic floor disorders in Kersa district Eastern Ethiopia: a community-based study. BMC Women S Health. (2018) 18:1. 10.1186/s12905-018-0585-1 29902997 PMC6003007

[66] Hasuda T Ueda A Wei CN. Prevalence of symptomatic pelvic floor disorders among Japanese women. J Women S Health Care. (2017) 06:1000389. 10.4172/2167-0420.1000389

[67] Reiner CS Williamson T Winklehner T Lisse S Fink D DeLancey JO. The 3D pelvic inclination correction system (PICS): a universally applicable coordinate system for isovolumetric imaging measurements, tested in women with pelvic organ prolapse (POP). Comput Med Imag Graph. (2017) 59:28–37. 10.1016/j.compmedimag.2017.05.005 28609701 PMC5526449

[68] Awan UA Guo X Khattak AA Hassan U Bashir S. HPV vaccination and cervical cancer screening in Afghanistan threatened. Lancet Infect Dis. (2023) 23:141–2. 10.1016/S1473-3099(22)00868-4 36610440

[69] Shoukat T Awan UA Mahmood T Afzal MS Wasif S Ahmed H. Epidemiology of toxoplasmosis among the Pakistani population: a systematic review and meta-analysis. Pathogens. (2022) 11:675. 10.3390/pathogens11060675 35745528 PMC9227424

[70] Bashir S Hussain M Ali Khan A Hassan U Mushtaq KS Hameed M. Renal transplant pathology: demographic features and histopathological analysis of the causes of graft dysfunction. Int J Nephrol. (2020) 2020:7289701. 10.1155/2020/7289701 33489373 PMC7787863

[71] Khattak AA Awan UA Nadeem MF Yaqoob A Afzal MS. Chloroquine-resistant Plasmodium falciparum in Pakistan. Lancet Infect Dis. (2021) 21:1632. 10.1016/S1473-3099(21)00700-3 34838225 PMC9760118

